# C:N:P Molar Ratios, Sources and ^14^C Dating of Surficial Sediments from the NW Slope of Cuba

**DOI:** 10.1371/journal.pone.0125562

**Published:** 2015-06-25

**Authors:** Guadalupe de la Lanza Espino, Luis A. Soto

**Affiliations:** 1 Instituto de Biología, UNAM México D.F., México; 2 Instituto de Ciencias del Mar y Limnología, UNAM México D.F., México; University of Illinois at Chicago, UNITED STATES

## Abstract

The surficial sediments recovered from 12 sites located near the channel axis of the Florida Straits and the lower slope off NW Cuba were analyzed for total organic carbon (TOC), nitrogen (TN), phosphorus (TP), elemental C:N:P ratios, C and N isotopic values, and ^14^C dating. The depth profiles of TOC, TN, and TP (0-18 cm) displayed a downcore trend and a significant variation. The TOC values were low (0.15 to 0.62%; 66 to 516 µmol g^-1^). Sites near the island’s lower slope had lower TOC average concentrations (158-333 µmol g^-1^) than those closer to the channel axis (averaging 341-516 µmol g^-1^; p <0.05). The TN concentrations near the lower slope attained 0.11% (80 µmol g^-1^), whereas, towards the channel axis, they decreased to 0.07% (55 µmol g^-1^; p<0.05). The C:N ratios ranged from 1.9 to 10.2. The mean molar C:N ratio (5.4) indicated a marine hemipelagic deposition. The TP was lower at sites near the lower slope (38.4 to 50.0 µmol g^-1^; 0.12% to 0.16%) than those near the channel axis (50.0 to 66 µmol g^-1^; 0.15 to 0.21%). C:P fluctuated from 7.7 to 14.1 in the surficial sediment layer. The bulk organic δ^13^C_org_ and δ^15^N values confirmed pelagic organic sources, and the ^14^C dating revealed that the sediments were deposited during the Holocene (1000-5000 yr BP). We suggest that the hydrodynamic conditions in the Straits influence vertical and advective fluxes of particulate organic material trapped in the mixed-layer, which reduces the particulate matter flux to the seabed.

## Introduction

The carbon, nitrogen and phosphorus contents and their molar ratios in sea-water and marine sediments are regularly used to determine the origin and transformation of organic matter (OM). These ratios can be influenced by a series of environmental factors highlighted by climate, terrigenous input, bathymetry, and bottom circulation [1–9]. There is evidence that organic C and N contents display decreasing trends in sediments along depth gradients (200 to 3000 m), such as off the Mississippi River Delta in the Gulf of Mexico [3]. In this region, the sedimentary OM derived from the river runoff is more depleted in N and P than that from a marine source. Interestingly, the C:N ratio in the Sargasso Sea and the South Atlantic exhibit increasing depth trends [6]. These examples serve to illustrate the relevance of regional environmental conditions in the composition of sinking particles of organic matter (POC).

In the Straits of Florida, the deep sea floor in the southern sector near Cuba has remained unexplored for several decades. Current initiatives associated with the search for fossil fuels in the seabed have renewed interest in the study of deep sea processes, such as the erosion of surficial sediments, new sedimentological depositional models, organic particle fluxes, and benthic biodiversity [10–14]. A previous geophysical survey of the study area [15] indicated the presence of topographic features on the northwestern coast of Cuba, such as sediment mounds, numerous sink holes and knolls on the slope sea floor associated with collapsed karstic structures that may be indicative of a seeping oil and gas province [16]. In this complex scenario, the hydrodynamic conditions prevailing in the southern Straits of Florida may play an important role in the decomposition and digenetic processes of sedimentary organic matter. The present study is focused on the quantification of the organic C and N and the total N and P contents in surficial sediments obtained from 12 deep sites distributed among three Blocks leased to REPSOL-YPF Cuba S.A. within the NW Exclusive Economic Zone (EEZ) of Cuba. This research identifies potential sources of organic matter based on the sediments’ molar ratios (C:N) and their δ^13^C and δ^15^N values. Similarly, radiocarbon dating was used to determine the sediments’ ages and to infer the sedimentation rate in the study area.

## Materials and Methods

**Fig 1:**
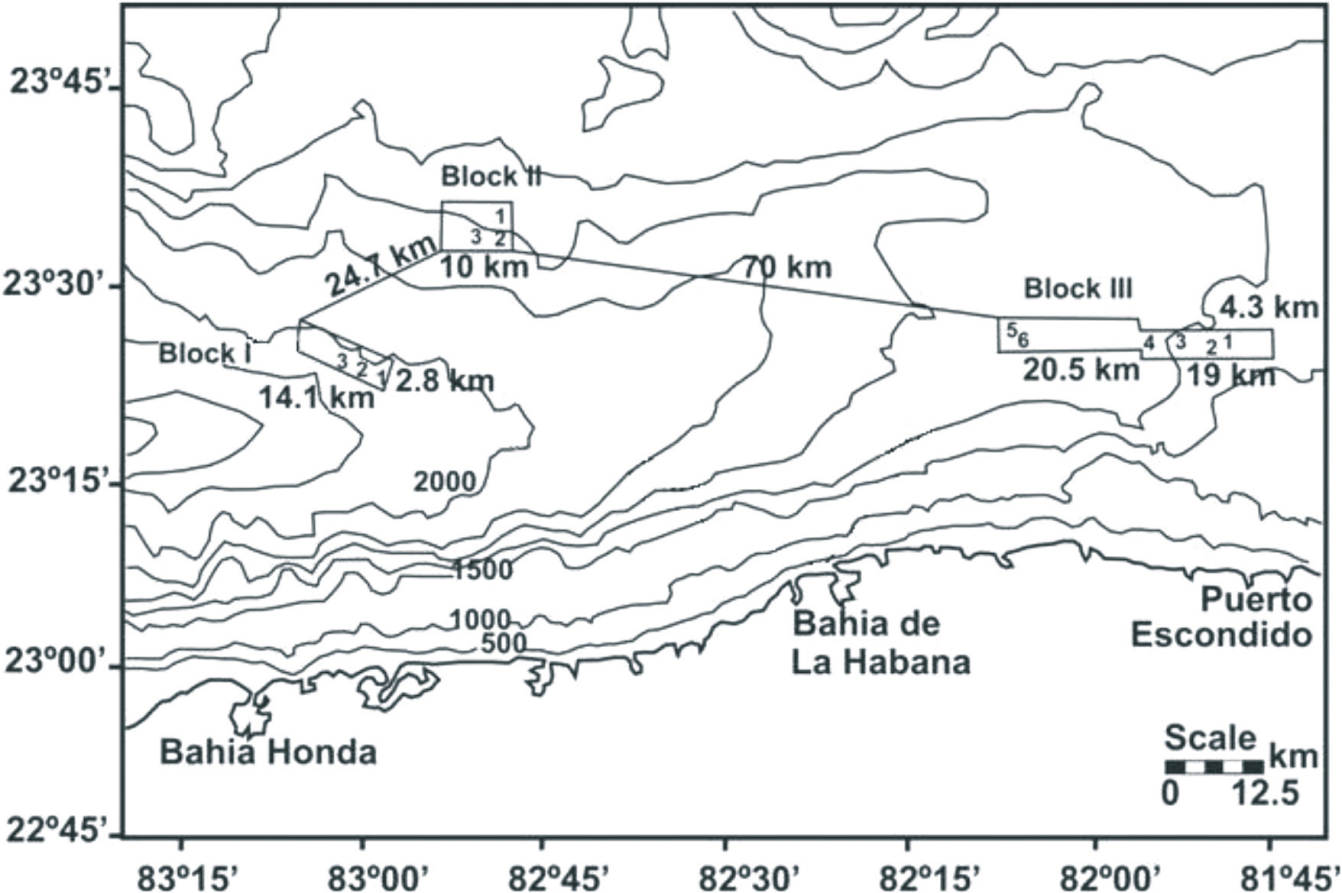
Bathymetry. Bathymetry of the NW slope of Cuba, Southern Florida Straits. The explored Blocks I, II and III are indicated from left to right, including their dimension, the location of the 12 sites sampled in the Blocks and the distance between them (modified from CESIGMA, 2002).

The study area included 12 sites on the seabed of the insular slope of the southwestern channel of the Florida Straits (between 23° 23’ 57” N, 83° 06’ 47” W and 23° 40’ 39” N, 81° 44’ 37” W) off of Bahía Honda and Puerto Escondido (Fig 1). The sampling sites were distributed among three preselected Blocks referred to as I (three sites), II (three sites), and III (six sites). Near-surface sediments were sampled with a Reineck box-corer (0.06 m^2^). The recovered box-cores were subcored with a 6 cm diameter and 30 cm long fiberglass core-liner. The subcores were freeze-dried and later divided into 4 cm sediment depth intervals. The resulting sediment fractions were lyophilized and ground. The organic carbon (OC) determinations involved oxidation with K_2_Cr_2_O_7_ and H_2_SO_4_, processing the excess of dichromate with Fe(NH_4_)_2_(SO_4_)_2_ (0.5 N) and diphenylamine as an indicator [17] with the modification of Loring and Rantala [18]. The total nitrogen (TN) was determined by a Kjeldahl digestion following Jackson [19] methodology and applying a spectrophotometric analysis suggested by Rodríguez-Medina [20]. For this purpose, to approximately 0.5 g of sediment it was added 1g of K_2_SO_4_, 0.5 g of selenium, 5 ml of concentrated H_2_SO_4_. The sample was heated-up at 360° C for 2h to obtain (NH_4_)_2_SO_4_ and then was transferred to 1000 ml of bi-distilled water solution, adjusting the pH at 7; NH_4_ was determined through the formation of indophenol blue whose concentration was measured in a spectrophotometer Coleman Junior II Mod -620 (analytical precision 0.005 μM/L) at 630 nm and expressed in μM per gram of sediment. The organic nitrogen (ON) was calculated from the correlation between total TN vs. organic carbon (TOC) recommended by Calvert [7]. A significant intercept in the N axis (42 μmol g^-1^) allowed the estimation of the ON by subtracting the N_inorg_ from the TN.

The total phosphorus (TP) was determined according to Holm’s [21] procedure, later modified by Rodríguez-Medina [20]. Approximately 120 mg of sediment was pre-digested with 9 ml of a mixture of 250 ml of H_2_SO_4_, 200 ml of HNO_3_ and 550 ml of water and kept under 1 kg of pressure for 2 h. To the resulting digested sediment sample it was added 100 ml of bi-distilled water, adjusting the pH at 7; a subsample diluted 1:10 was then prepared with (NH_4_)_2_MoO_4_ and ascorbic acid to form the phospho-molybdate. This complex of blue coloration was measured in a Coleman Junior II Mod-620 spectrophotometer (analytical precision of 0.01–0.03 μM/L) at 880 nm and transformed to μM per gram of sediment. The concentrations of OC, TN, and TP were expressed as μmol g^-1^ and transformed to percentages for discussion purposes. The C:N:P, C:N and C:P ratios were calculated based on their respective molecular weights. Assuming that nitrogen may be present in the form of nitrate or ammonium in clay minerals, it was estimated that TN mostly corresponded to organic nitrogen (ON). With this assumption, the C/N ratio (TOC/TN) was calculated as an indication of the source of organic matter (OM) in the sediments [22, 23].

Bulk surficial sediment samples were obtained for isotopic analysis (δ^15^N and δ^13^C_org_). The sediment samples were acidified with 1 N HCl bath for 24 h to eliminate carbonates in the sediment matrix, washed with distilled water and oven dried overnight at <80°C. The dry samples were ground to a fine powder with a mortar and pestle. Isotopic analysis was performed with an elemental analyser coupled online via a Finnigan-MAT 252 Stable Isotope Ratio Mass Spectrometer (analytical precision ± 0.2 ‰) using air nitrogen and PDB standards for N and C, respectively. The δ^13^C and δ^15^N values are reported relative to the VPDB (Vienna Pee Dee Belemnite) and air nitrogen standards (international reference materials: IAEA N1, USGS 25 and 26), respectively, using δ (delta) notation and parts per thousand (‰). The X-RAY diffraction (XRD) analyses of sediment subsamples were conducted with a Philips 1130/96 diffractometer utilizing Cu K_α 1, 2_ radiation directed toward randomly oriented samples. Standard scans were recorded from 4° to 70° (2 θ) at 2°/min. The radiocarbon ages of OC from 1 g of dry near-surface sediments subsampled at levels 0–4, 4–8, and 8–12 cm from Block II (site 3) were determined by UNAM’s Radiocarbon Laboratory (LUR:UNAM-1377). The radiocarbon-derived sedimentation rates (ω) were estimated for each level as ω = Z (cm)/T kyr^-1^, where Z is sedimentary depth and T is conventional age (years before present, BP).

Statistical analysis was performed with SYSTAT 9 software and the significance level α was set to α = 0.05 in all test. All data sets were determined to have a non-normal distribution. Due to the randomized design and unequal sample number of the data set, intergroup differences of carbon and nitrogen values recorded in the three Blocks, non-parametric methods (Kruskal-Wallis with tied ranks and Friedman’s test) were used. Similarly, linear regression analyses were applied to establish a correlation among sedimentary variables (TOC, TN, TP)

### Description of the Study Area

The three preselected Blocks located in Cuba’s Exclusive Economic Zone (Fig 1) extended across the slope of the southern Straits of Florida. This area, which separates Cuba, the Florida Peninsula and the Bahama Bank, is the deepest area (>1600 m) of a trough-like feature recognized as a graded axial slope that shallows eastwards and northwards [24, 25]. The insular margin is extremely steep and rugged. Block I, located at a depth of 2169 m off Artemisa Province, Cuba, had a surface of 28.49 km^2^, comprising a v-shaped valley oriented along the central axis of the channel, with faulting structures and small canyons on its western end. Block II (54.11 km^2^), at 1640 m, exhibited an escarped surface and a faulted region running from NE to SW. These two Blocks were separated by 27.7 km. Block III (174.32 km^2^), at 1650 m, included a ridged bottom with prominent knoll-like structures protruding *ca*. 100 m above the seabed. This third Block, located approximately 70 km due east from the other two, lay closer to the insular shelf (<28 km), just off Puerto Escondido, Mayabeque Province, Cuba. The three examined Blocks are part of the planktonic-foraminiferal biofacies recognized in the Florida-Bahama area [26]. The XRD analysis of the sediment samples revealed a mineral composition dominated by calcite and aragonite with a minor proportion of fluorapatite. Allochems were bioclasts (foraminifera tests and microshell fragments) embedded in a fine-grained calcite matrix (foram biomicrite).

### Hydrographic conditions

In the present study, the flow of bottom water had a SEE direction and an average velocity of 4 cm·s^-1^, slightly higher than the 3 cm·s^-1^ observed in abyssal basins [27]. Numerical current models [28] have revealed a complex and variable flow field in the Yucatan Channel. The Loop Current’s interannual variability and eddy shedding have been invoked to explain such variability, which seems to be confined to the top 1000 m. These hydrodynamic conditions may exert a significant influence on the vertical and advective fluxes of particulate organic material (POC), which can be trapped in the mixed-layer (~200–700 m) in the Straits, thus reducing the downward flux of POC to the seabed. In contrast, the thermohaline structure at the bottom of the southern Straits was stable at 5 ml·l^-1^ O_2_, 3 − 4°C, and 35.0 psu.

## Results

### Total Organic Carbon (TOC)

The OC concentration in the three Blocks exhibited a decreasing organic pool with increasing bottom depth. The values ranged from 158 to 666 μmol g^-1^, with averages of 406, 497 and 279 μmol g^-1^ for Blocks I, II and III, respectively (Table 1). The 12 analyzed subcores displayed a significant decline in TOC in the top 12 cm, except for Blocks I and II, in which the presence of discrete dark banding at 7 and 9 cm was detected. Interestingly, the first two Blocks, lying close to the main axis of the Straits channel, have slightly enriched TOC values relative to the six sites of Block III located at the toe of the slope. Nonetheless, the TOC contents among the 12 examined sites showed significant differences (ANOVA f = 0.001), which were attributed to a lower sediment flux rate of OM in Block III.

**Table 1:** Elemental composition.

	Depth	TOC	TN	TP	C:N:P	TOC:TN	TOC:TP	δ^13^C_org_	δ^15^N
	cm	μmol g^-1^	μmol g^-1^	μmol g^-1^	Molar ratios	Molar ratios	Molar ratios	‰	‰
BLOCK I (1)	0–4	383	61.0	49.7	7.7:1.2:1	6.3	7.7		
	4–8	358	53.8	51.1	7.0:1.1:1	6.6	7.7		
	8–12	408	78.2	48.9	8.4:1.6:1	5.2	8.4		
**AVERAGE**		**383**	**64.4**	**49.9**		**6.0**	**7.9**		
**± Std.**		**25**	**12.1**	**1.1**		**0.7**	**0.4**		
								-18.8	+5.6
BLOCK I (2)	0–6	383	61.6	52.8	7.3:1.2:1	6.2	7.3		
	6–12	383	62.8	48.4	8.0:1.3:1	6.1	8.0		
	12–18	358	69.4	65.9	5.4:1.1:1	5.2	5.4		
**AVERAGE**		**375**	**64.6**	**55.7**		**5.8**	**6.9**		
**± Std.**		**14**	**4.2**	**9.2**		**0.6**	**1.3**		
								-18.5	+5.4
BLOCK I (3)	0–6	450	75.0	55.5	8.1:1.4:1	6.0	8.1		
	6–12	425	70.7	88.5	4.8:0.8:1	6.0	4.8		
	12–18	358	62.9	54.9	6.5:1.2:1	5.5	6.5		
**AVERAGE**		**411**	**69.6**	**66.3**		**5.8**	**6.5**		
**± Std.**		**47**	**6.2**	**19.2**		**0.3**	**1.7**		
								-18.7	+5.7
BLOCK II (1)	0–5	500	64.3	49.2	10.2:1.3:1	7.7	10.2		
	5–10	400	48.2	51.1	7.9:0.9:1	8.3	7.9		
	10–15	375	46.6	50.6	7.4:0.9:1	8.1	7.4		
	15–20	325	73.7	49.7	6.5:1.5:1	4.4	6.5		
**AVERAGE**		**400**	**58.2**	**50.1**		**7.1**	**8.0**		
**± Std.**		**73**	**13.2**	**0.9**		**1.8**	**1.6**		
								-18.5	+5.2
BLOCK II (2)	0–6	325	50.5	51.4	6.3:0.9:1	6.4	6.3		
	6–12	350	41.5	52.5	6.6:0.8:1	8.4	6.6		
	12–18	350	74.5	57.4	6.1:1.3:1	4.7	6.1		
**AVERAGE**		**341**	**55.5**	**53.7**		**6.5**	**6.3**		
**± Std.**		**14**	**17.0**	**3.2**		**1.9**	**0.3**		
								-18.9	+6.4
BLOCK II (3)	0–4	666	46.1	52.8	12.9:0.9:1	14.1	12.9		
	4–8	408	39.4	48.4	8.7:0.8:1	10.4	8.7		
	8–12	475	71.1	65.9	9.7:1.5:1	6.7	9.7		
**AVERAGE**		**516**	**52.2**	**55.7**		**10.4**	**10.4**		
**± Std.**		**134**	**16.7**	**9.2**		**3.7**	**2.2**		
								-18.7	+5.7
Block III (1)	0–4	158	56.9	39.5	4.0:1.4:1	2.8	4.8		
	4–8	158	54.9	36.7	4.4:1.5:1	2.9	4.4		
	8–12	158	43.4	39.0	4.1:1.1:1	3.6	4.1		
**AVERAGE**		**158**	**51.8**	**38.4**		**3.1**	**4.4**		
**± Std.**		**0**	**7.3**	**1.5**		**0.4**	**0.4**		
								-18.7	+3.6
BLOCK III (2)	0–5	158	61.3	28.6	5.5:2.2:1	2.6	5.5		
	5–10	158	59.9	36.3	4.4:1.7:1	2.6	4.4		
	10–15	66	57.7	35.7	1.9:1.5:1	1.6	1.9		
**AVERAGE**		**127**	**59.6**	**33.5**		**2.3**	**3.9**		
**± Std.**		**52**	**1.8**	**4.5**		**0.6**	**1.8**		
								-19.1	+5.8
BLOCK III (3)	0–5	358	90.2	45.3	7.9:2.0:1	4.0	7.9		
	5–10	258	82.8	50.0	5.2:1.7:1	3.1	5.2		
	10–15	325	63.4	51.4	6.3:1.2:1	5.1	6.3		
**AVERAGE**		**313**	**78.8**	**48.9**		**4.1**	**6.5**		
**± Std.**		**50**	**13.8**	**3.2**		**1.0**	**1.4**		
								-18.7	+5.7
BLOCK III (4)	0–5	383	85.1	50.0	7.7:1.7:1	4.5	7.7		
	5–10	358	82.3	48.9	7.3:1.7:1	4.4	7.3		
	10–15	258	75.3	51.1	5.1:1.5:1	3.4	5.1		
**AVERAGE**		**333**	**80.9**	**50.0**		**4.1**	**6.7**		
**± Std.**		**66**	**5.1**	**1.1**		**0.6**	**1.4**		
								-18.6	+5.7
BLOCK III (5)	0–6	291	79.8	50.0	5.8:1.6:1	3.7	5.8		
	6–12	325	72.2	47.8	6.8:1.5:1	4.5	6.8		
	12–18	358	64.7	48.4	7.4:1.3:1	5.5	7.4		
**AVERAGE**		**325**	**72.3**	**48.7**		**4.6**	**6.7**		
**± Std.**		**33**	**7.6**	**1.1**		**0.9**	**0.8**		
								-18.8	+5.6
BLOCK III (6)	0–6	325	72.4	46.7	7.0:1.6:1	4.5	7.0		
	6–12	258	58.2	47.3	5.5:1.2:1	4.4	5.5		
	12–18	158	54.4	47.8	3.3:1.1:1	2.9	3.3		
**AVERAGE**		**247**	**61.7**	**47.3**		**3.9**	**5.3**		
**± Std.**		**83**	**9.5**	**0.6**		**0.9**	**1.9**		
								-18.6	+5.4

### Stable Carbon Isotope Analysis

**Fig 2:**
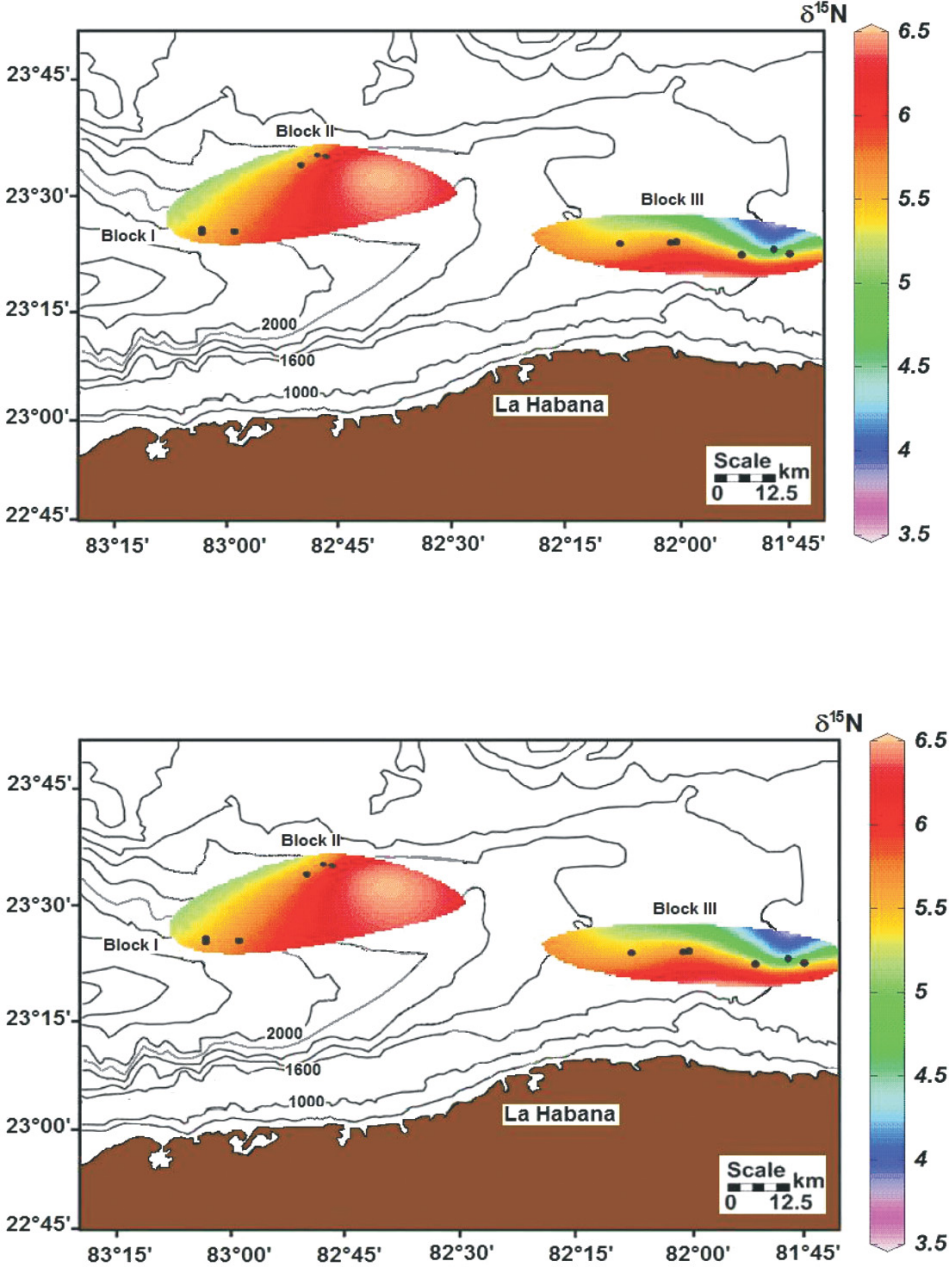
δ^13^C_org_ and δ^15^N. Spatial distribution of δ^**13**^C_**org**_ and δ^**15**^N values in surface sediments collected from the three Blocks examined in the Southern Florida Straits off the insular slope of the NW coast of Cuba.

The bulk δ^13^C_org_ values of surface sediments had a narrow range in all Blocks (-18.5 to -19.1 ‰), with an average value of -18.7±0.17 ‰ (Table 1), which corresponds to an oceanic organic carbon source. However, a non-parametric statistical test (Kruskal-Wallis) with tied ranks rejected the assumed equality of δ^13^C_org_ values among the Blocks (0.05<p<0.010). Regardless of the proximity of the sites from which the core samples were recovered, the δ^13^C_org_ values exhibited a small spatial variability (Fig 2). A small enrichment gradient is observed along the seabed from the westernmost Blocks (I and II) towards the toe of the slope (Block III).

### Total Nitrogen (TN)

The concentrations of TN downcore exhibited a dissimilar trend in the twelve analyzed subcores, particularly in Block III. The values ranged from 39.4 to 90.23 µmol g^-1^, with an average of 64 μmol g^-1^. All subcores displayed a heterogeneous pattern between 5 cm and 12 cm. Contrary to the OC spatial trend, Blocks I and II near the Straits’ channel axis revealed mostly low TN values (0.05% and 0.1% TN), whereas Block III featured the highest TN concentrations, particularly in the subcores obtained at sites 3, 4 and 5, located at the base of the Cuba’s basin (1600 m) (Table 1).

### Stable Nitrogen Isotope Analysis

The surficial sediment samples from the Southern Straits of Florida had mostly enriched δ^15^N values, ranging from +3.6 to +6.4 ‰ (Table 1), with an average of +5.4±0.7 ‰. The δ^15^N values in the deeper Blocks (I and II) were relatively homogeneous, whereas in Block III (Fig 2), variability occurred due to the presence of significantly smaller depleted values (+3.6 ‰). The equality of the Blocks’ δ^15^N values was rejected (Friedman’s test p <0.368). Based on the estimated δ^15^N average recorded here (+5.4±0.7 ‰), a significant input of pelagic organic matter was inferred.

### TOC:TN ratios

The estimation of TOC:TN molar ratios added further support to the sedimentary differences observed among the three Blocks. For instance, in Block III, the lowest values of TOC:TN (2.8 to 4.5) were recorded in the top 4 and 6 cm sediment fractions, though no clear downcore trend was observed (Table 1).

The highest TOC:TN ratios corresponded to Block II near the channel axis. Ratios of 7.7 to 14.4 were recorded between 4 and 6 cm, with a tendency to decrease to 4.4 to 6.7 below 8 cm (Table 1). This was attributed to an increase in TN rather than a decrease in TOC. Block I exhibited more homogeneous downcore TOC:TN ratios, which progressively decreased from 6.0 to 6.3 in the top 4 or 6 cm, down to ratios of 5.2 to 5.5 below 8 cm.

The TOC:TN ratios were markedly different between Block III (toe of the slope) and Blocks I and II (channel axis). In Block III, the ratios oscillated between 1.6 and 5.5 in the uppermost centimetres, with a heterogeneous downcore trend. These low ratios were presumably caused by lower TOC and higher TN contents. In contrast, in most of the subcores of Blocks I and II, the TOC:TN ratios were significantly higher (between 6.4 and 14.1) in the uppermost centimetres and progressively decreased with depth (Table 1).

**Fig 3:**
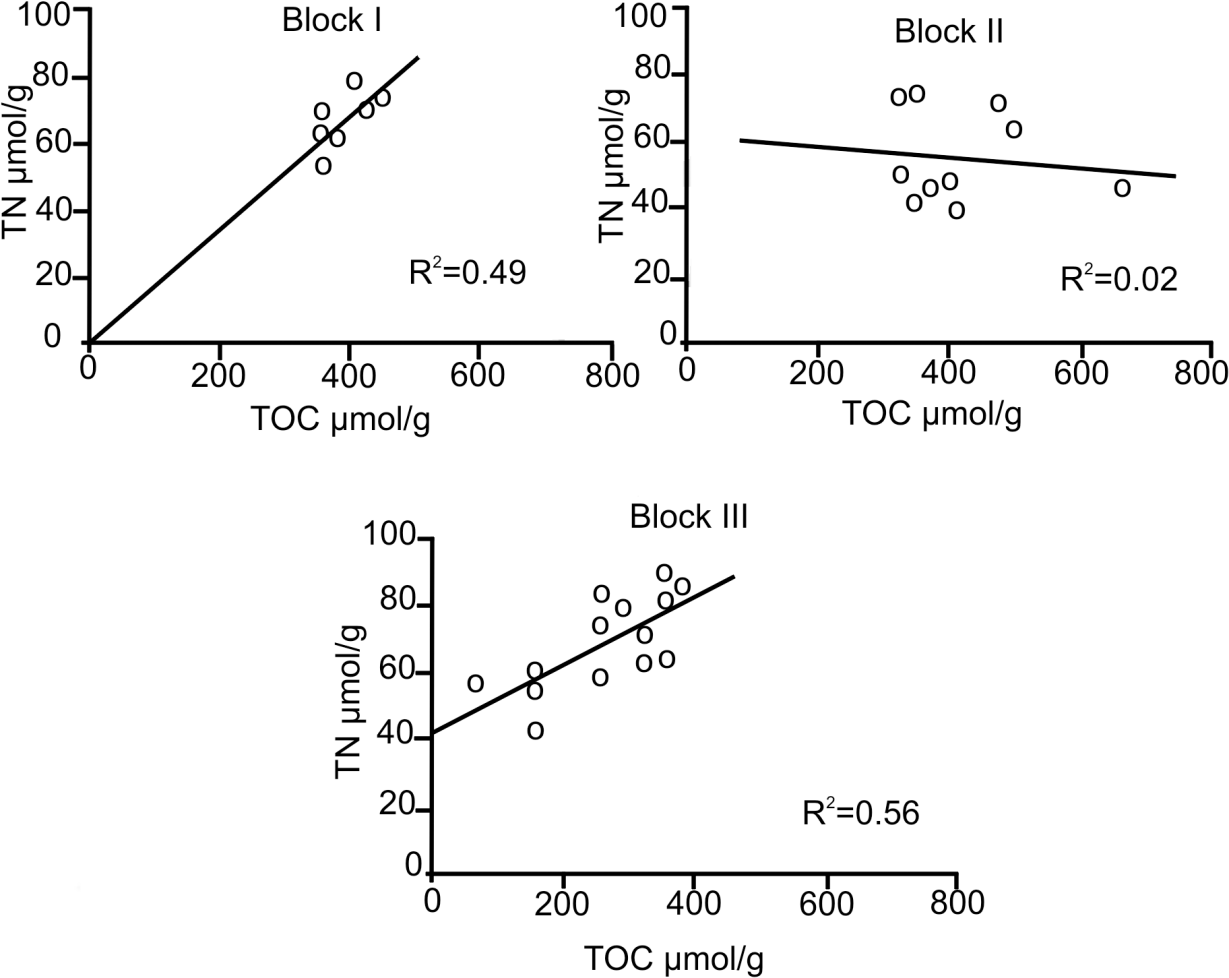
TOC:TN ratios. Correlations of the TOC:TN ratios of each Block examined on the NW slope of Cuba, Southern Florida Straits.

In view of the reverse trends of TOC and TN recorded among the three Blocks, the corresponding correlations were calculated. These correlations are useful in determining the contribution of inorganic nitrogen (IN) to the organic matter pool [2, 29]. By fitting a line by least-squares linear regression in the TN vs TOC plots of the three Blocks, significant differences among the Blocks were highlighted (Fig 3). Block I had a fair linear correlation (R^2^ = 0.49), with a zero intercept for the two variables, which was caused by the significant dominance of organic nitrogen within the TN pool, based on the applied calculations [7]. In Block II, the TN vs TOC plot displayed highly dispersed values with no correlation. The plot for Block III had the best-fitted line and a significant correlation (R^2^ = 0.56), with an intercept at 42 μmol g^-1^ on the TN axis (Fig 3). To assess the organic N contained in the sedimentary TN pool, 42 μmol g^-1^ of inorganic nitrogen were subtracted from each value [7]. Following this procedure, it was estimated that the quantity of ON contained in the TN pool of the subcores from Block III fluctuated from 26% to 53% in the top 4 cm. The observed downcore trend of decreasing ON was attributed to the preferential remineralization over that of the organic matter pool (Table 2).

**Table 2:** Estimation of the organic carbon and organic nitrogen.

Block III	Depth	TOC	ON	TOC:ON	TON of TN	OP	OC:OP
	cm	μmol g^-1^	μmol g^-1^		%	μmol g^-1^	
Site 1							
	0–4	158	14.9	10.6	26.0	8.6	18.4
	4–8	158	12.9	11.9	23.0	5.7	27.7
	8–12	158	1.4		3.3	8.0	19.8
	**AVERAGE**		**9.7**			**7.4**	**22.0**
	**± Std.**		**7.2**			**1.5**	**5.0**
Site 2							
	0–5	158	19.3	8.2	31.0		
	5–10	158	17.9	8.8	29.0	5.3	29.9
	10–15	66	15.8	4.2	27.0	4.7	14.2
	**AVERAGE**		**17.7**			**3.3**	**22.1**
	**± Std.**		**1.8**			**2.9**	**11.1**
Site 3							
	0–5	358	48.2	7.4	53.0	14.3	25.1
	5–10	258	40.8	6.3	49.0	19.0	13.6
	10–15	325	21.4	15.2	38.0	20.4	15.9
	**AVERAGE**		**36.8**			**17.9**	**18.2**
	**± Std.**		**13.8**			**3.2**	**6.1**
Site 4							
	0–5	383	43.1	8.9	51.0	19.0	20.2
	5–10	358	40.3	8.9	49.0	17.9	20.0
	10–15	258	33.3	7.8	44.0	20.1	12.9
	**AVERAGE**		**38.9**			**19.0**	**17.7**
	**± Std.**		**5.1**			**1.1**	**4.2**
Site 5							
	0–6	291	37.8	7.7	47.0	19.0	15.3
	6–12	325	30.3	10.7	42.0	16.8	19.4
	12–18	358	22.7	15.8	35.0	17.4	20.1
	**AVERAGE**		**30.3**			**17.7**	**17.7**
	**± Std.**		**7.6**			**1.1**	**3.4**
Site 6							
	0–6	323	30.4	10.7	42.0	15.7	20.6
	6–12	258	16.2	14.2	28.0	16.3	15.9
	12–18	158	12.4	12.8	22.0	16.8	9.4
	**AVERAGE**		**19.7**			**16.3**	**15.3**
	**± Std.**		**9.5**			**0.6**	**5.6**

The average ON concentrations in Block III oscillated between 9.73 and 38.90 μmol g^-1^. A significant ON enrichment (average = 35.3 μmol g^-1^; 0.05%) was recorded at three sites (3, 4 and 5) located in Cuba’s basin. The remaining sites in this Block featured mostly low ON values that were one fold less than the above average. Factors such as bioturbation, different sedimentation rates and oxidation of OM may be invoked to account for the observed differences among the sites. In site 2 of Block III, we determined that 0.02% was equivalent to 19.32 μmol g^-1^ (in the first 4 cm) and that 0.06% was equal to 43.12 μmol g^-1^ in a nearby site (site 4). In this Block, we noted a predominance of IN (47 to 74% in the first 6 cm), which increased with depth (Table 2). This finding was further corroborated by the TON:TN correlation (R^2^ = 0.99), which indicated the presence of 50% IN contained in sediments of Block III.

**Fig 4:**
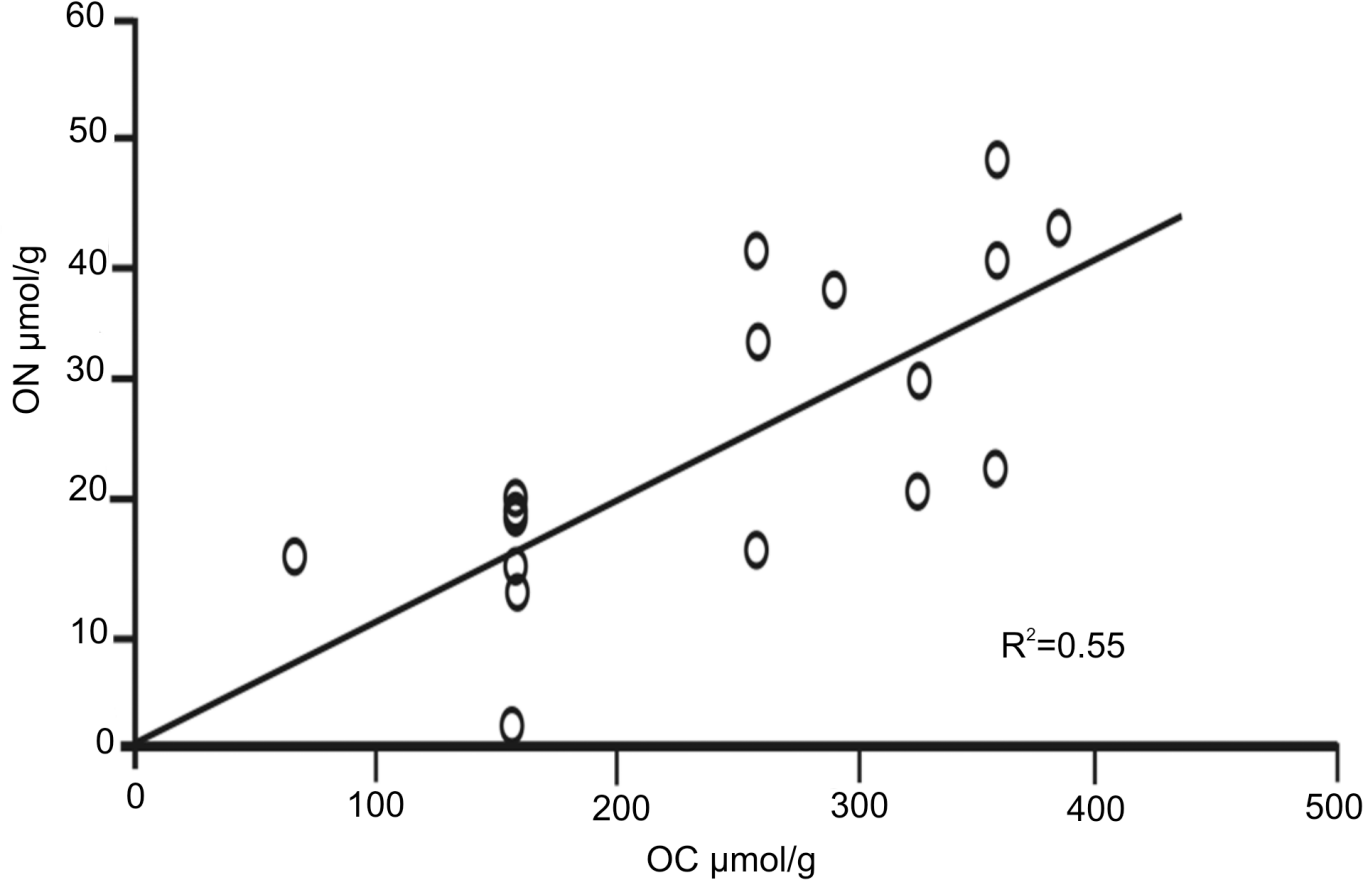
TOC:ON ratios. Correlation of the TOC:ON ratios in subcores from six sites of Block III on the NW slope of Cuba, Southern Florida Straits.

A substantial increase in the TOC:ON ratio was observed in Block III when the calculations included the subtraction of the IN percent from the nitrogen pool (Tables 1 and 2). However, no definite tendency was distinguished among all sites sampled in this Block. A significant correlation (R^2^ = 0.55) was obtained between ON and OC (Fig 4), involving the predominance of IN, possibly in the form of NH_4_ [30].

### Total Phosphorus (TP)

The TP concentration values recorded in the three examined Blocks did not display a distinct spatial distribution. A similar condition was also distinguished along the depth gradient (f = <0.0013). In spite of the variability observed in the TP values obtained from Blocks I and II, no significant differences were detected among the sites. However, these values were slightly more enriched than those recorded in Block III (Table 1).

**Fig 5:**
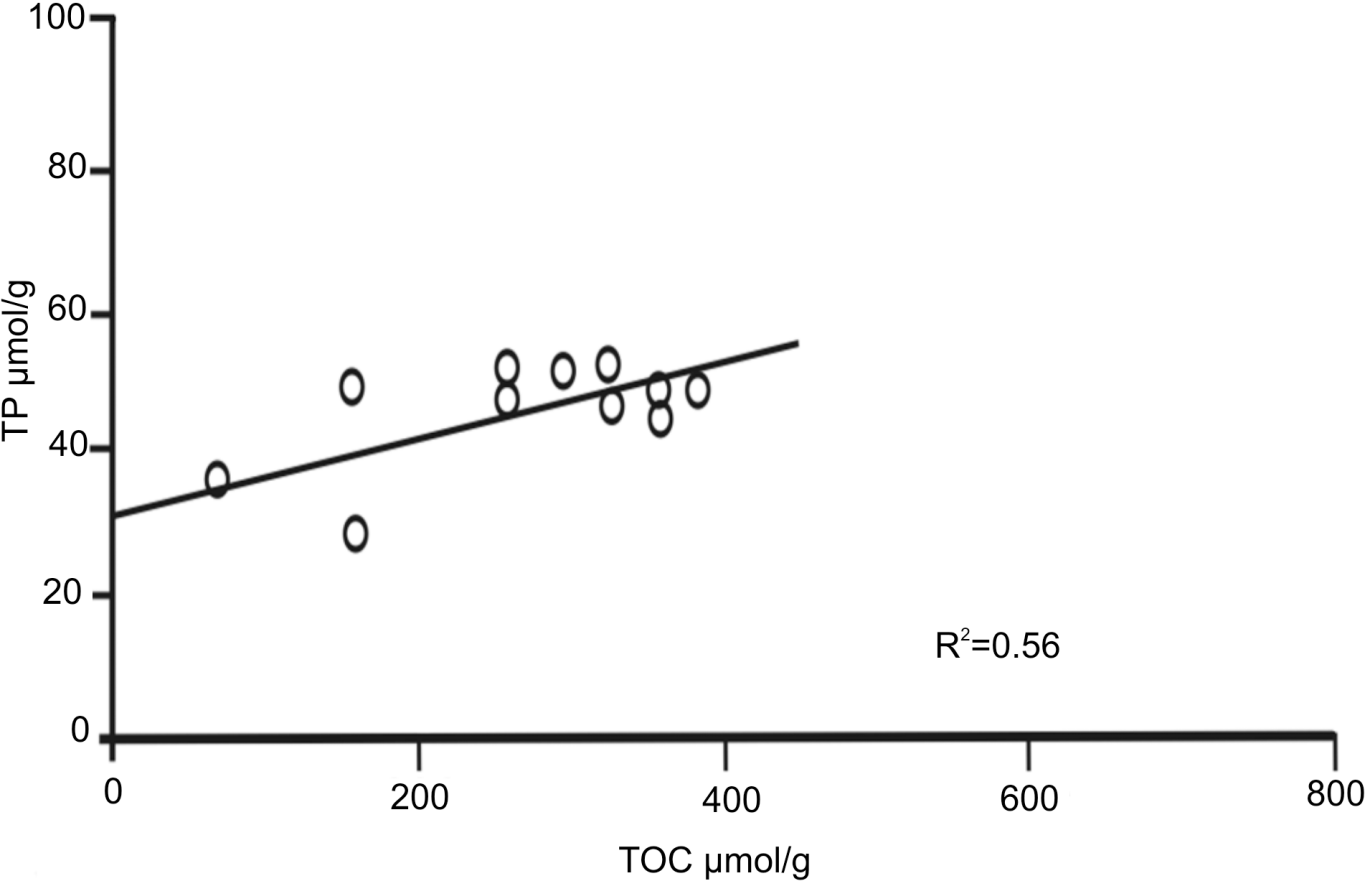
TOC:TP ratios. Correlation of the TOC:TP ratios in subcores from six sites of Block III on the NW slope of Cuba, Southern Florida Straits.

The individual correlations of TP and TOC obtained for each Block were not significant, with the exception of Block III (R^2^ = 0.56). In this particular case, the intercept on the TP axis was 31 μmol g^-1^, which corresponded to the inorganic phosphorus (IP) calculated through the subtraction recommended by Calvert [7] (Fig 5). In general, IP predominated in all the surficial sediments of the three Blocks.

**Fig 6:**
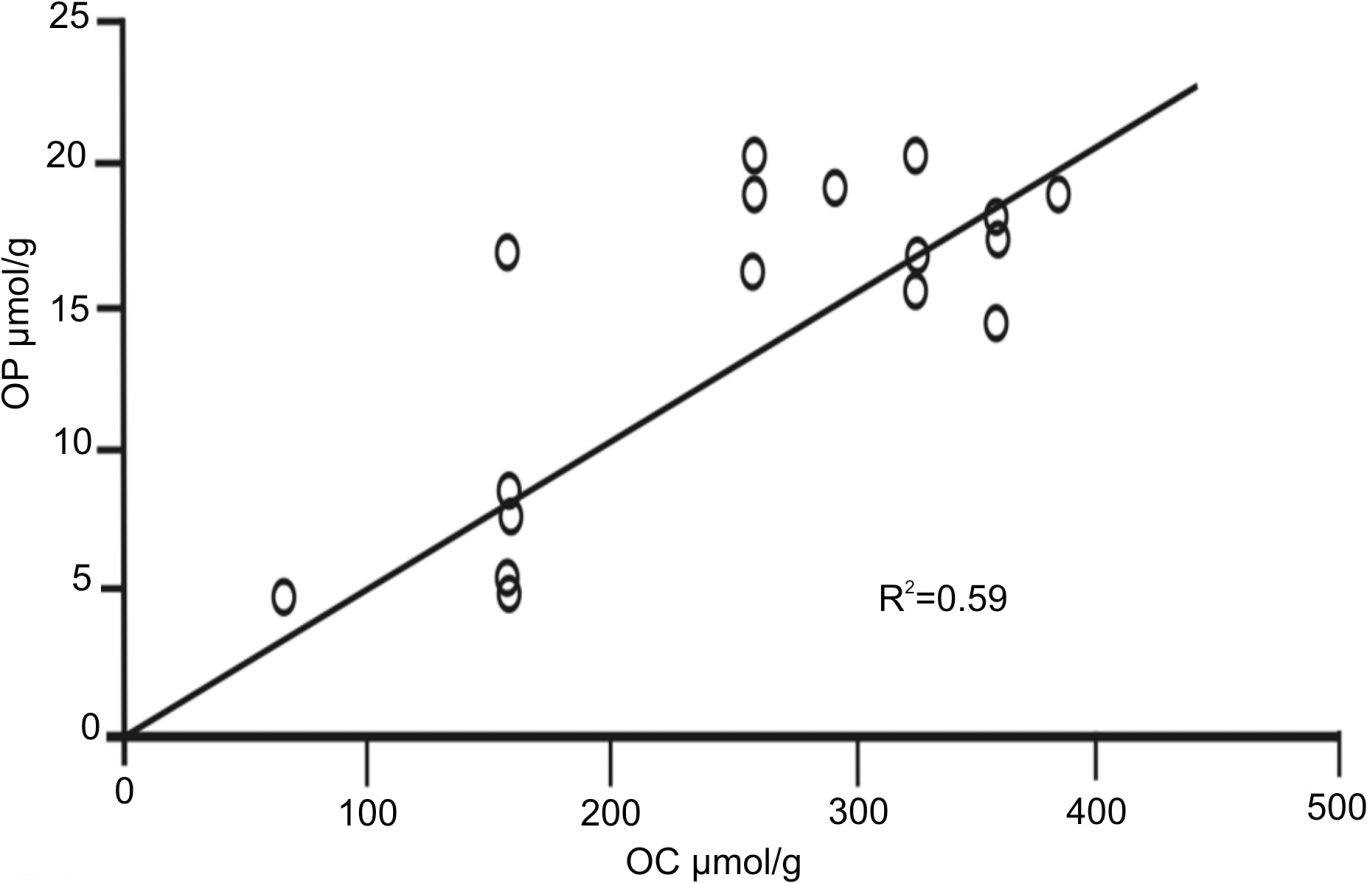
OP:TOC ratios. Correlation of the OP:TOC ratios in subcores from six sites of Block III on the NW slope of Cuba, Southern Florida Straits.

In the present study, organic phosphorus (OP) accounted for 10 to 38% of the TP, with concentrations ranging from 3.3 to 19.0 μmol g^-1^ (Table 2). No visible downcore trend in OP was detected in Block III. At sites 4, 5, and 6 of this Block, the OP:TOC ratios were significant (R^2^ = 0.59), suggesting the presence of OP, even at 18 cm (Fig 6). In the case of Blocks I and II, there were no significant correlations.

The TOC:TP ratios varied from 1.9 to 12.9 (Table 1), and the values were rather heterogeneous in the three Blocks. This ratio tended to decrease with depth due to the increase of TP in most of the Blocks.

### ^14^C Ages of Surficial Sediments

The radiocarbon determination of the sedimentary carbonate, mainly foraminifera and micromollusks, from core 3 of Block II revealed that the sediments are modern and belong to the Subboreal and Subatlantic Chronozone of the Holocene epoch (~ 1000–5000 radiocarbon years BP). The top two sediment layers (0–4 cm and 4–8 cm) exhibited small differences in conventional ages (years before present, BP), in contrast with those recorded at 8–12 cm, which encompassed sediments that are older by almost two orders of magnitude (Table 3).

**Table 3:** Calibrated ages and ranges.

Block II core 3	Z cm	^14^C age yr BP (1δ)	Cal age ranges (2 δ)
LUR: UNAM-1377	0–4	2280 ±70	Cal BP 1223–1511
LUR: UNAM-1378	4–8	2760±70	Cal BP 1708–2068
LUR: UNAM-1379	8–12	4915 ±70	Cal BP 4423–4798

**Fig 7:**
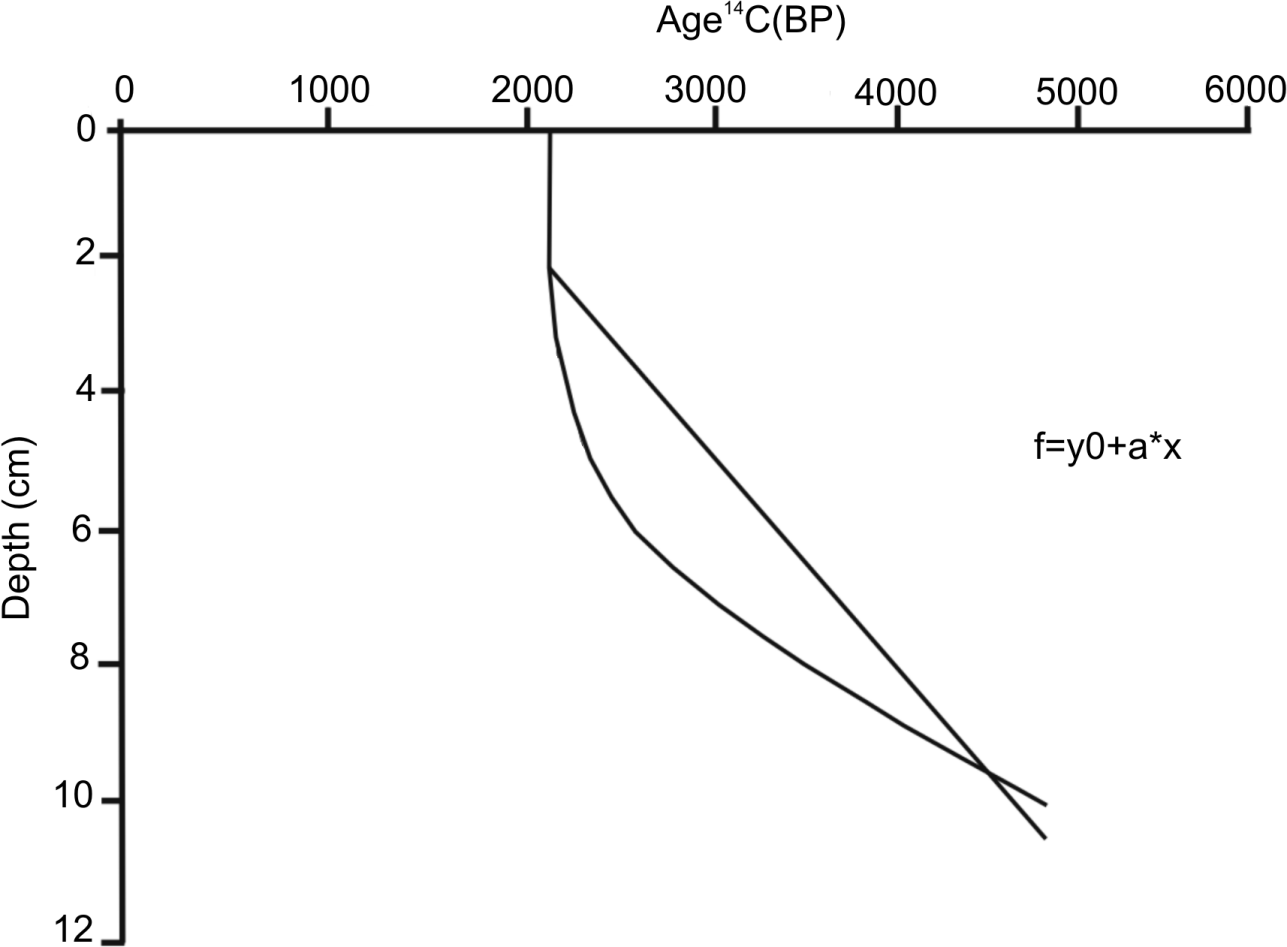
Conventional age. Conventional age (years before present, BP) and best fitting line of surface sediments from Block II on the NW slope of Cuba, Southern Florida Straits.

Bioturbation caused by macroinfauna above the mixed sediment layer (SML) (~4–6 cm) most likely promoted the reworking of the sediment in the subsurface layers. Sediment dating using ^14^C in the carbonate fraction yielded low sedimentation rates, ranging from 1.0 cm Kyr^-1^ near the surface to 2.04 cm Kyr^-1^ in subsurface levels (Fig 7), resulting from OC enrichment at approximately 5000 yr BP.

## Discussion

The two dark subsurface horizons of organic material detected in the sediment subcores of Blocks I and II possibly resulted from episodic events of deposition of terrigenous material exported by surrounding environments, as suggested by Malloy and Hurley [25]. Echeverría-Rodríguez et al. [31] noted that the terrigenous material detected in offshore seismic lines may act as a seal above hydrocarbon-generating Tertiary rocks. In deep cores obtained from a transect made along the western Great Bahama Bank, the marl/limestone alternations observed in sediments from the basin were indicative of high-frequency sea-level changes [32]. The intercalation of light-colored limestones and dark-colored mud bands may also be a reflection of turbidites deposited during sea-level low stands. However, as demonstrated in this study by the ^14^C sediment dating, the sampled subsurface sediments show signs of an OC enrichment that took place nearly 5000 yr BP, during Holocene’s Subboreal Chronozone [33].

According to Santschi and Row [34], radiocarbon dates provide a good test for long-term sedimentation rates in near-surface sediments. Presumably, in the area of study, the POC flux to the seabed must be low given the hydrodynamic conditions in the Southern Straits of Florida [14]. Our estimated sedimentation rate (2.2 kyr^-1^) approaches that of the Sigsbee Abyssal Basin of 2.7 kyr^-1^ [33] for sediments dated to 5380±65 yr and is also similar to sediment rates reported for the abyssal floor of the NE Atlantic [35].

The estimated averages of OC in Blocks I and II were similar to the weight percent organic carbon reported for two sites in the Sigsbee Abyssal Plain (0.49 and 0.55 wt. % OC) [36]. In contrast, the third Block encompassed mostly lower OC values in a predominantly calcareous sedimentary environment. The inverse relation between the concentrations of C_org_ and calcium carbonates has been observed in other parts of the world as the Arabian Sea and the sea of Japan [37]. In other regions directly exposed to terrigenous input, such as the Amazon’s fan and the Mississippi River Delta [2,3], similar weight percentages of OC (0.34% to 0.57%) have been recorded at depths between 1000 and 2000 m. In fact, the TOC % values here recorded in the Cuban margin fall within the concentration profiles of the C_org_ per cent known for the US Atlantic Coast (200–2000 m) included in the global distribution of marine sediments [37]. However, in the area studied here, the surface waters are known for their oligotrophic conditions, attaining values of 50–200 mg C m^-3^ d^-1^ in the euphotic zone [38,39], and hence, the vertical organic carbon flux to the sea floor must be low. This would explain the impoverished sedimentary organic carbon in our subcore samples. Oxic organic matter degradation cannot be ruled out to account for the OC decrease. This degradation process is responsible for the OC decay in surficial sediments from the eastern subtropical Atlantic near the Canary Islands (1360 m) [4].

The average δ^13^C_org_ value of -18.7±0.17 ‰ estimated here approaches that known for surficial sediments from the continental shelf of South Florida (-18.5±0.7‰) [40]. This isotopic value corresponds to autotrophic organic carbon synthesized by phytoplankton, whose range is -18.0 to -24.0 13‰, with a fairly constant average of -21.013‰ [41]. The bulk δ^13^C_org_ values recorded here appear more negative in comparison with the value (-16.8 ‰) recorded for sedimentary organic materials deposited on the slopes of the Great Bahama Bank (in the lee of Andros Island) [42]. This is most likely caused not only by diagenesis but also by the predominance of isotopically light pelagic-derived carbon over the flux of isotopically heavier platform-derived sediments from the Cuban carbonate margin.

**Fig 8:**
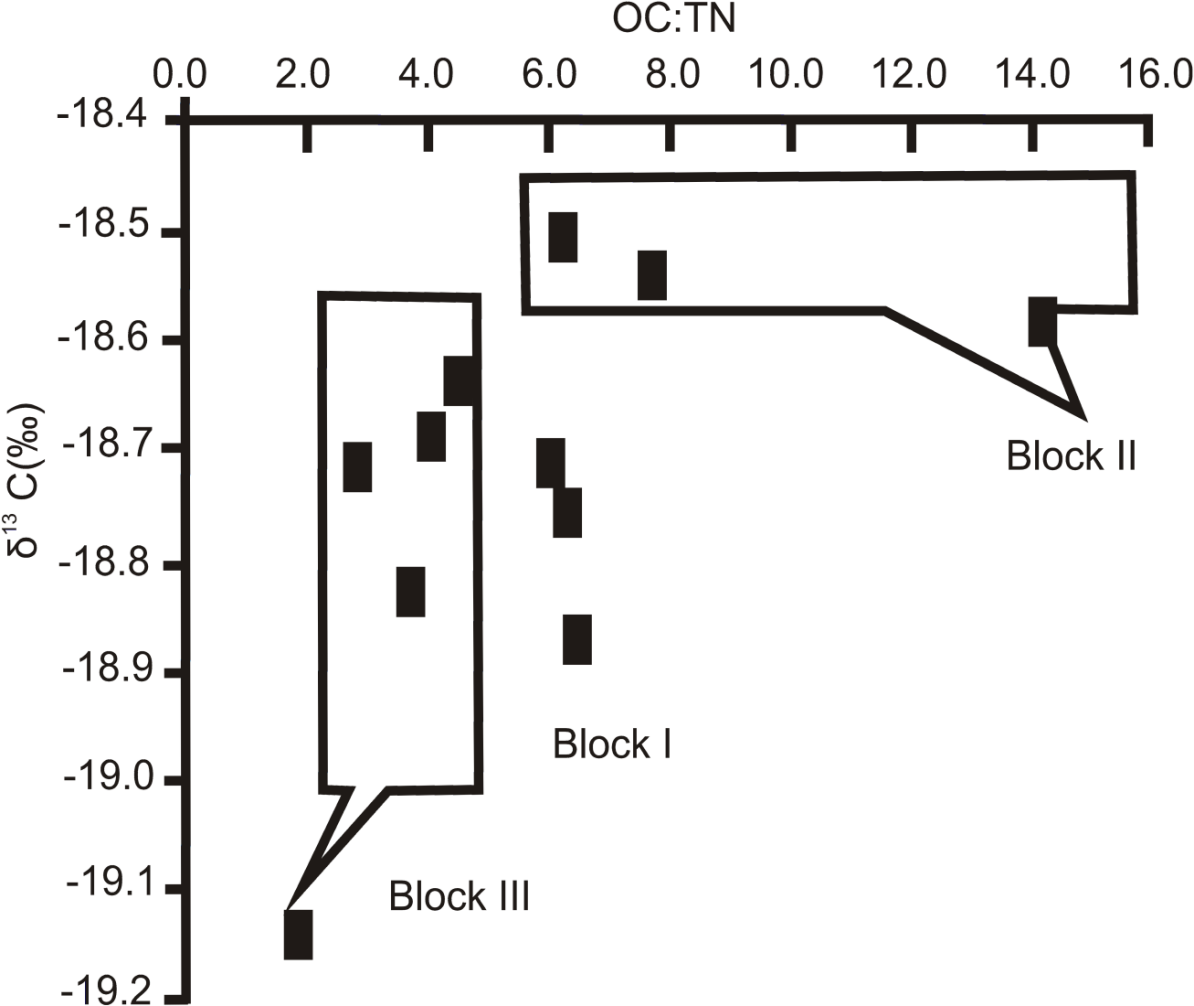
δ^13^C (‰) values and OC:TN. Relationship between δ^**13**^C (‰) values and OC:TN ratios for the three Blocks located on the NW insular slope of Cuba.

An additional indicator of the origin of the organic matter is the C:N ratio [37,43]. C:N ratios > 15 are indicative of terrestrial input while C:N < 8 favour marine input. The mean molar C:N ratio in the sedimentary organic matter from three sites in each examined Block ranged from 2.4 (Block I) to 3 (Block II) and up to 5 (Block III). These ratios are indicative of labile organic matter being deposited by a marine hemipelagic source [14]. The dark core-banding detected below 10 cm in the subcore obtained from site 1 in Block II produced a higher OC:TN ratio, which was possibly caused by episodic terrestrial inputs in the area studied, as referred to earlier (Fig 8).

The equivalent weight percentage of TN (0.12%) of the maximum concentration recorded here (90.23 μmol g^-1^) falls below the percentage of 0.17% calculated in the northwestern Gulf of Mexico at 1470 m, where presumably the sedimentary rate is 0.005 cm/yr [3]. The abyssal environment of the Gulf of Mexico is known for its low TN concentration, which is limited to 0.08% [3]. In our study, most of the low TN values obtained from the upper 10 cm of subcores belonging to Blocks I and II (average of 60.73 μmol g^-1^) corresponded to less than 0.09 weight percentage of total nitrogen, somewhat similar to that previously reported for the Gulf [3].

In comparison with the δ^15^N value of +3.6 ± 0.1‰ for the sediments from the continental shelf of South Florida [40], just north of the studied area, our values were relatively higher by approximately 1.8 ‰. Due to the similar nitrogen isotope composition of coastal and oceanic particulate matter, this enrichment likely reflects the organic matter decomposition of material sinking to greater depths. In the slope waters of the NW Gulf of Mexico, the δ^15^N composition of nitrate has an average of 5.0 ‰ and of 5.5‰ in N particles in the mixed-layer [44]. The positive signature of the sedimentary nitrogen isotope measured the NW Cuban margin suggests that nitrate diffusing from below the thermocline may be taken up by plankton [45] that ultimately sinks to the bottom to become part of the hemipelagic sediments that characterize this area [46]. Interestingly, our estimated δ^15^N average (+5.4±0.7‰) falls within the range of that reported for zooplankton (+5.9±0.7‰) [40], which slightly emphasizes the incorporation of pelagic POC into the sediments without fractionation. Presumably, the absence of isotopic fractionation in marine sediments can be attributed to the dominance of a marine component in the sedimentary organic matter along with a slight fraction of the terrestrial component [8].

TOC:TN ratios, usually, maintain a decreasing trend from 7.6 to 5 in superficial sediments from the continental shelf to deeper regions [1,3]. However, this gradient may be inconsistent with the progressive dilution of terrigenous OM in the open sea [1]. It is clear that OM may have different sources and can also be altered by diagenesis processes in its transport to deeper regions [1]. Additionally, the preferential degradation of organic compounds rich in nitrogen may also play an important role in this process.

As in the case of the C_org_, the TN % values recorded from surficial sediments of the Cuban margin followed a similar trend. They seem also impoverished ranging from 0.06 to 0.1%. This coincides with the organic nitrogen percent reported from the slope regions of the Gulf of Mexico and the eastern seaboard of the US [37]. The TN pool in the studied area showed signs of a decrease in ON, possibly caused by bioturbation, sedimentation rates and oxidation of OM. These same factors were responsible for a significant reduction in ON (0.026 to 0.006%) in surficial sediments (0.5–1 to 11–12 cm) along a transect in the Central Equatorial Pacific Ocean [1]. The estimated IN in this region (12 to 64%) [1] exhibited a much larger range than that recorded in sediments from the NW Cuban slope (47 to 74%). In contrast, in the Gulf of Mexico, the IN ranges from 20 to 50% [2]. Several authors have suggested that sedimentary IN fluctuations could be the result of NH_4_ adsorbed by clay [4,47,48]. This condition seems to be present in estuarine and shelf sediments off South China [49], where almost 39% of the IN fraction is in the form of NH_4_.

The availability of carbon and nitrogen sources, the oxidation conditions, and the diagenesis of the organic matter pool may play an important role in the OC:ON ratios. When the OC:ON ratios of Block III were recalculated, the resulting values were higher than those reported by Redfield et al. [50] but fell within the range of those published by several authors [1,5,6,8,29,51].

TP tends to decrease with bottom depth. For instance, in the northern Gulf of Mexico, just off the Mississippi Delta, TP values ranged from 33.67 to 18.23 μmol g^-1^ at depths from 200 to 2000 m, indicating different chemical weathering conditions along the depth gradient [2]. Similarly, in the Ulleung Basin, Korea, the TP values feature a depth-dependent decreasing trend from 35.5 to 13 μmol g^-1^, attaining the lowest values at 1000 m. However, the most heterogeneous and highest values were recorded below 2000 m [52].

On the slope of NW Cuba, the maximum TP values corresponded to the deepest sites in Block I (2160 m), and a small decrease was detected towards sites sampled in Blocks II and III (~ 1640 m). Processes such as burial flux of phosphorus and sedimentation rates may play an important role on the TP concentrations observed here. There are at least four factors that may influence the ability of the sediments to retain P [53]. These factors vary as a function of the depositional environment. However, the predominance of IP in all the examined subcores can be correlated to the presence of authigenic carbonate fluorapatite (CFA). The formation of this phosphate mineral enhances the retention of P produced by OM degradation and the reductive dissolution of ferric iron phases embedded below the redoxcline [53]. It has been suggested that OP (in sediments 50 cm deep) is the main form of phosphorus in recent sediments, representing 40% of the TP, and also constitutes the major source of the carbonate fluorapatite that precipitates as depth increases [52].

In a sediment core obtained off the Mississippi Delta (110 m), most of the TP was contained in three authigenic phases, i.e., CFA, biogenic apatite and the CaCO_3_ reservoir [53]. Apparently, the increasing trend of authigenic P downcore is caused by the formation of authigenic CFA at the expense of OP. As indicated earlier, such a trend was not observed in the present study. An interesting finding was that the TP concentrations in some sites of Block I (2169 m deep) were almost threefold higher (average >66.3 μmol g^-1^) than those off the Mississippi Delta at 2250 m (18.23 μmol g^-1^) [43]. Perhaps the low OC:TP ratios in pelagic sediments may also depend on the extended degradation of detrital OM, which eventually leads to the formation of enriched residual refractory phosphorus. This condition seems to be present in Block III, in which different sedimentation and burial processes that enhance remineralization may have promoted the formation of refractory residual phosphorus. The OC:OP values between 62 and 54 determined for depths of 1000 and 2000 m off the Mississippi Delta [44] are significantly higher than those of Block III (Table 2). However, such ratios from the northern Gulf of Mexico were considered abnormally low for pelagic sediments and reflected the presence of organic matter residues enriched with OP refractory compounds [1]. In Block III, the maximum OC:OP ratio attained a value of 29.9 and was attributed to high OP (Table 2). Several reasons are offered to justify the variations in the OC:OP ratios of organic matter buried in marine sediments. First, different sedimentation rates can cause systematic variations in such ratios. Second, differences in the inputs of either terrestrial plants or marine phytoplankton with distinct C signatures [54] could also cause variations.

The OC:OP and OC:TN ratios (Tables 1 and 2) of Block III dropped below those given by Redfield et al. [50] for marine plankton. These conditions are present in the deep waters of the Gulf of Mexico [2] and suggest bacterial N and P enrichment as a possible cause. However, in the specific case of the OC:TN ratios, the preferential absorption of IN may explain the observed values in the Gulf of Mexico, a circumstance that may also occur in the calcareous sediments of the insular slope of NW Cuba.

## Conclusions

Despite the relative spatial proximity of the three Blocks studied here, their sedimentary compositions, in terms of carbon, nitrogen and phosphorus components, exhibited marked differences, which were attributed to diagenetic processes acting during the transport of organic compounds to the seabed. In general, the analyzed subcores can be classified as organic-poor slope carbonate sediments, in which terrestrial input is negligible and from a region characterized by its low surface productivity. Indeed, the hydrodynamic conditions prevailing in the oligotrophic waters of the Straits of Florida must exert a significant influence upon the vertical and advective fluxes of POC trapped in the mixed-layer, thus causing a low OM input to the bottom. The OC:ON elemental ratios, as well as the δ^13^C_org_ isotopic values, of surficial sediments confirmed the deposition of labile OM from a marine hemipelagic source.

The values of TN in Blocks I and III were also low and featured a direct correlation with the concentration of OC. The differences detected among the sites and the low concentrations observed were interpreted as results of a preferential degradation of organic compounds rich in nitrogen. The δ^15^N values added further support to the incorporation of pelagic POC to surficial sediments with a reduced fractionation. In Block II, located near the channel axis (~ 1600 m), certain subcores displayed dark subsurface banding (8–18 cm depth) that most likely resulted from episodic terrigenous input events occurring during the Holocene, reflected in subtle TN and TP increments in the vertical profiles.

The applied TOC vs. TN correlation revealed the predominance of IN within the N pool in Block III. Factors such as bioturbation, different sedimentation rates and oxidation of OM were invoked to explain the significant ON enrichment detected in sites of this Block located near the insular slope. However, in other sites within Block III, 47 to 74% of the TN in the top 6 cm of the subcores corresponded to the IN fraction and was possibly adsorbed by clays in the form of NH_4_.

No definite trend was recognized in the TP concentration values obtained from the twelve subcores analyzed. The significant spatial and depth-related heterogeneity in TP was ascribed to the burial flux of phosphorus and to different depositional conditions. All examined sites feature a predominance of IP due to the presence of authigenic carbonate fluorapatite (CFA). This mineral was detected in the solid phase by X-ray diffraction analysis and represented the third most abundant component, after calcite and aragonite.
